# Symptom clusters in cancer patients: a review of the literature

**DOI:** 10.3747/co.2007.145

**Published:** 2007-10

**Authors:** G. Fan, L. Filipczak, E. Chow

**Keywords:** Symptom cluster, cancer

## Abstract

Cancer patients often experience multiple symptoms, and those symptoms can independently predict changes in patient function, treatment failures, and post-therapeutic outcomes. Symptom clusters are defined as two or more concurrent symptoms that are related and may or may not have a common cause. The purpose of the present study was to review, in cancer patients, common symptom clusters and their predictors.

Using medline, embase, Cochrane Central, and cinahl, we conducted a literature search on symptom clusters in cancer patients. Studies that investigated predetermined clusters were not included. We identified seven individual studies and one group of five studies validating the M.D. Anderson Symptom Inventory. These studies had been published between 1997 and 2006. Two of the seven individual studies and the group of five studies that had validated the M.D. Anderson Symptom Inventory included patients with any cancer type; three studies included breast cancer patients only; and two studies included lung cancer patients only.

A gastrointestinal cluster consisting of nausea and vomiting was the single cluster common to two of the studies. The severity of this cluster increased when patients were treated with chemotherapy. No common clusters were found in the lung and breast cancer patient populations. However, breast cancer patients experienced more symptom cluster involvement while undergoing chemotherapy. We noted methodology disparities among the papers with regard to assessment tools used, statistical analyses, and populations.

Research on symptom clusters is still in an early stage. Multiple symptoms clearly affect prognosis, quality of life, and functional status. The study of symptom clusters is important for its implications regarding patient management, and a consensus on appropriate research methodology is vital.

## 1. INTRODUCTION

Cancer patients often experience multiple symptoms, and symptoms seldom occur in isolation in patients with advanced cancer. Cancer patients have been reported to experience an average of 11–13 concurrent symptoms 1,2. Symptoms may be a result of the disease itself or of the associated treatment. They may considerably affect the patient’s sense of wellbeing and his or her physical and social functions. However, most clinical studies in symptom research have focused largely on the treatment of individual symptoms. This focus has undoubtedly led to some advances in the understanding of particular symptoms, but patients seldom present with a single symptom—which may perhaps explain why treating one symptom may not necessarily improve quality of life.

The co-occurring symptoms that cancer patients often experience may or may not be interrelated. Although a continuing focus on single-symptom research is crucial, it is equally important that symptom management research begin to evaluate multiple symptoms in cross-sectional and longitudinal study designs. In addition, research needs to more closely examine the relationships between multiple symptoms, specific interventions, and patient outcomes.

The occurrence of multiple symptoms independently predicts changes in patient function, treatment failures, and post-therapeutic outcomes. Recently, systematic attention has turned to the occurrence of multiple symptoms in cancer patients, with reports that “symptom complexes” or “clusters” may occur. Dodd and colleagues were among the first to coin the term “symptom clusters” in their work with pain, fatigue, and sleep disturbances 3. Their definition of symptom clusters specifies the presence of 3 or more concurrent symptoms that are related and that may or may not have a common cause. They did not define the strength of the cluster relationship, however. Another paper described symptom clusters as 2 or more symptoms that are related, that occur together, that form a stable group, and that are relatively independent of other clusters 4.

Although the definition of a symptom cluster has not yet been fully elucidated, the presence of multiple co-occurring symptoms can have an adverse effect on patient outcome and may have a synergistic effect as a predictor of patient morbidity. The objective of the present review was to report patterns of symptom clusters found in cancer patients to date. The specific research questions were these:

- Do certain symptom clusters commonly occur in oncology patients?
- Do predictors of symptom clusters exist?

## 2. MATERIALS AND METHODS

In October 2006, we conducted a literature search in four databases (medline, embase, Cochrane Central, and cinahl) to identify all studies on symptoms clusters in cancer patients. The search was unrestricted as to date, but restricted to publications in English. The subject headings “symptom cluster,” “multiple symptoms,” “symptom constellation,” “symptom combinations,” or “co-occurrence of symptoms” were combined with “cancer” or “oncol-.”

The search identified a total of 223 articles. Citations and articles listed as “relevant” in PubMed were also considered for review. All abstracts, full-text publications, letters, and editorials were reviewed. Only studies that tested statistically for a symptom cluster within a set of symptoms were included. Articles that reported predetermined symptom clusters were excluded from this review.

## 3. RESULTS

Seven individual studies and one group of five studies validating the M.D. Anderson Symptom Inventory (mdasi) found evidence of symptom clusters in cancer patients 5–16. The publication dates ranged from 1997 to 2006. Identification of symptom clusters was the primary endpoint in five of the seven studies, but the study populations varied. Two individual studies and the group of five studies that validated the mdasi included patients with any type of cancer 5–11; three studies included only breast cancer patients ^(14–16)^; and two studies included only lung cancer patients ^(12,13)^.

### 3.1 Common Clusters in Cancer Patients

Identifying symptom clusters in patients with any cancer type was the primary endpoint for two of the papers 5,6 discussed in this section.

Walsh and Rybicki 5 found 7 symptom clusters in 922 patients with advanced cancer. Their 38-symptom checklist graded each symptom as absent, mild, moderate, or severe. Cluster analysis using an agglomerative hierarchical method with linking average was used to identify the clusters in this 1-centre study. Only 25 symptoms with >15% prevalence were selected for analysis, and a correlation of ≥0.68 was used to define the final clusters (fatigue/anorexia/ cachexia, neuro-psychological, upper gastrointestinal, nausea/vomiting, aerodigestive, debility, and pain). Table I lists the symptoms that belong in each cluster.

Chen and Tseng reported 3 symptom clusters in their study of 151 cancer patients in a Taiwan centre 6. Participants were asked to complete the 13-item mdasi and the 14-item Hospital Anxiety and Depression Scale. This cross-sectional study used principal component analysis with oblique rotation to factor the selected symptoms. The 3 identified clusters (sickness, gastrointestinal, emotional) were found using the foregoing statistical technique, which explained 55% of the variance (Table I). Mean scores for the sickness syndrome were higher when patients reported pain or were advanced in their disease. Patients undergoing chemotherapy treatment experienced higher mean scores for the gastrointestinal cluster.

During validation of the English, Japanese, Chinese, and Filipino versions of the mdasi, the authors found 2 consistent symptom clusters in all four translations of the assessment tool 7–10. The sample populations ranged in number from 206 to 527 patients, and all four studies used the principal axis factor analysis technique with oblimin rotation for cluster determination. The first cluster was labelled general symptom severity (comprising pain, fatigue, disturbed sleep, emotional distress, shortness of breath, drowsiness, dry mouth, sadness, difficulty remembering, and numbness or tingling). The second cluster was labelled gastrointestinal (comprising nausea and vomiting). In three of the validation studies (English, Chinese, Filipino), patients receiving chemotherapy had significantly higher mean scores in the gastrointestinal cluster (*p* < 0.05, *p* < 0.002, *p* < 0.005 respectively). The Japanese version did not report chemotherapy as a factor influencing the severity of symptoms within the gastrointestinal cluster.

Interestingly, the Russian validation of the mdasi found 3 clusters: general, treatment-related, and affective 11. There was no evidence of the gastrointestinal cluster. However, patients receiving chemotherapy experienced greater symptom prevalence.

The gastrointestinal cluster (labelled nausea/vomiting in the paper by Walsh and Rybicki) is the single cluster that is common to the reported studies. This cluster includes 2 symptoms: nausea and vomiting. When patients were treated with chemotherapy, the severity of the symptoms in the gastrointestinal cluster increased, as reported by Chen and Tseng and the authors of the English, Chinese, and Filipino validations of the mdasi.

Among the seven papers discussed here, no other common symptom clusters emerged. Notable are the differences in the methodology of the studies. Chen and Tseng and the authors of the mdasi validations used the same statistical method and assessment tool, but Walsh and Rybicki adopted a different statistical method and assessment tools.

### 3.2 Clusters in Lung Cancer Patients

Two studies included only lung cancer patients. Sarna and Brecht 12 were among the first authors to cluster symptoms. The primary endpoint of their study was to explore the structure of symptom distress in women with advanced lung cancer. They used the Symptom Distress Inventory to survey 60 women with advanced lung cancer who were receiving palliative treatment. To identify symptom combinations, the authors used principal component analysis with varimax rotation. Their results showed symptoms clustered in 4 groups explaining 63.3% of variance. These clusters were emotional and physical suffering, gastrointestinal distress, respiratory distress, and malaise. Table II lists the symptoms within each cluster.

Gift *et al.* 13 surveyed 220 newly diagnosed patients with lung cancer. These authors used the 37-item Physical Symptom Experience tool with a 3-point system to rate symptom severity. Because of missing responses, only 32 of the 37 symptoms were included in the analysis. Factor analysis was used to find and extract clusters, and only 1 cluster was found for which all symptoms were consistently correlated (Cronbach α = 0.73). The symptoms in that cluster were nausea, fatigue, weakness, appetite loss, weight loss, altered taste, and vomiting. Patients receiving chemotherapy experienced more symptoms in that cluster.

Because Gift *et al.* reported only 1 large symptom cluster, extracting a similar cluster from the symptom clusters found by Sarna and Brecht is difficult. Again, the differences in the methodologies used are notable. The limited research conducted thus far into symptom clusters in lung cancer patients makes it impossible to further explore common symptom clusters in this population.

### 3.3 Clusters in Breast Cancer Patients

Three studies included breast cancer patients only, but each study used different subpopulations. Bender *et al.* 14 explored differences in symptom clusters across 3 disease stages: early stage (*n* = 40, group 1), stage i/ii/iii (*n* = 88, group 2), and stage iv (*n* = 26, group 3). All patients completed multiple assessment tools including the Profile of Mood States, the Symptom Checklist, a daily symptom diary, the Menopausal Quality of Life Scale, and the Functional Assessment of Cancer Therapy Anemia/Fatigue scale. Using hierarchical cluster analysis in this exploratory secondary analysis, 4 clusters were found in group 1, 3 in group 2, and 2 in group 3. The 4 clusters seen in group 1 were fatigue, perceived cognitive impairment, mood problems, and other. The 3 clusters in group 2 were fatigue, perceived cognitive impairment, and mood problems. Group 3 patients experienced only the fatigue and mood problems clusters. Based on the definition that symptom clusters involve 2 or more symptoms, the fatigue and mood problems symptom clusters were found to be common across all 3 groups. Table III lists the symptoms in each cluster.

In a study that aimed to explore the occurrence and frequency of menopausal symptoms, Glaus *et al.* 15 found 1 symptom cluster in breast cancer patients (*n* = 373; 301 early-stage, and 72 late-stage) actively receiving hormone treatment. The Checklist for Patients with Endocrine Therapy and the International Breast Cancer Study Group Linear Analogue Scales for patients with endocrine treatment were used. The cluster analysis grouped symptoms using the agglomerative hierarchical method. The identified symptom cluster was named menopausal (comprising hot flashes/sweats, tiredness/fatigue, weight gain, vaginal dryness, and decreased sexual interest). The symptoms were found to be more prevalent in early-stage breast cancer patients.

Ridner 16 compared quality of life and symptoms in breast cancer patients with and without lymphedema. Each group accrued 64 patients who completed a 52-item symptom checklist developed by the authors, the 11-item Iowa 11 by 3 short-form Center for Epidemiologic Studies of Depression scale (to measure depressive symptoms), and the 37-item Profile of Mood States–Short Form to measure mood disturbances. Using analysis of covariance, a symptom cluster was found in women who had previously been treated for lymphedema. The cluster consists of alteration in limb sensation, loss of confidence in body, decreased physical activity, fatigue, and psychological distress.

In these studies, breast cancer patients experience common symptoms, but no common symptom clusters. Because each study examined different patient populations and used different assessment tools specific to the population under study, identification of common clusters would not be expected.

### 3.4 Predictors for Symptom Clusters

In the reported studies, a pattern in the occurrence of symptom clusters is emerging. Patients undergoing chemotherapy treatment and patients in the early stages of breast cancer tend to experience more symptom cluster involvement.

Chen and Tseng and the mdasi validation authors found that chemotherapy patients experience nausea and vomiting (symptoms in the reported gastrointestinal symptom cluster) at higher intensity levels. Similarly Gift *et al.* found that lung cancer patients experience a greater number of symptoms when treated with chemotherapy. These findings are consistent with the literature and entirely unsurprising, because nausea and vomiting are reported as two of the most distressing symptoms experienced by chemotherapy patients 17,18.

Bender *et al.* found more symptom clusters in early-stage breast cancer patients, and Glaus *et al.* found evidence that early-stage breast cancer patients are more inclined to experience symptoms in the menopausal symptom cluster. These findings must be interpreted cautiously because of confounding factors. For example, the bodily aches and pain experienced by patients in Bender *et al.*’s early-stage group may have been caused by recent surgeries. And the discrepancy in the sample sizes of the early- and late-stage patients in the study by Glaus *et al.* may have contributed to the results noted by the authors.

These findings are preliminary because of the lack of studies on symptom clusters completed to date and the lack of consistency in methodology between the studies that have been done. Once symptom cluster research is more developed, further research to find predictors of firmly established symptom clusters can be explored.

## 4. DISCUSSION

Symptom cluster research is in its early stages, and many questions remain unanswered in this field. Because the research thus far is limited, it is too early to confirm or deny the existence of common symptom clusters in cancer patients.

Some publications on symptom clusters have involved the symptoms depression, pain, sleep disturbance, and fatigue, but many of those studies did not statistically test to validate the symptom cluster 19–21; instead, the symptom clusters in those studies were derived based on prior knowledge and literature about the relationships between the chosen symptoms. As interest in symptom clusters begins to expand, it will be important to clearly define “symptom cluster” and to reach consensus on a methodology for data collection and analysis in symptom cluster research.

The literature search in the present review clearly shows disparities in the methodology for symptom cluster research. Symptom definitions, assessment tools, and statistical methods varied widely in the articles reviewed. Symptoms are subjective by nature, involve multiple dimensions, and are most often rated by the patient. Barsevick *et al.* 22 suggested that, because of the compound quality of symptoms, measuring only one dimension of a symptom would be a practical approach. In addition, they suggested that the ideal measure of symptom clusters would be consistent in the response scaling of the symptoms and in the measurement of parallel dimensions of each symptom within the same time period and clinical setting, and would implicate a reasonable response burden to the patient 22. These propositions are practical and can serve as useful guidelines in the design of symptom cluster research.

Still, it remains unclear when symptoms should be considered valid for analysis. Miaskowski *et al.* 23 delineated this issue and asked whether a specific cutoff score in symptom severity is warranted or whether the presence or absence of the symptom suffices in symptom cluster analysis. The answer to this question first requires consideration of the statistical method that is most compatible with the assessment tools used to discover indications of symptom clusters.

To maintain consistency of analysis in symptom cluster research, a single statistical method should be used. Researchers have used several statistical methods to derive symptom clusters, including factor analysis and cluster analysis. In the present review, two individual studies and the mdasi validation studies used factor analysis (principal component analysis with rotation), one individual study used factor analysis without rotation, three studies used cluster analysis, and one study used analysis of covariance.

Different methods may yield different sets of clusters, and results may vary depending on the statistical analysis technique used. Factor analysis examines the relationships between variables such as symptom severity and can make use of principal component analysis to explain covariance and test for group differences 22,24. On the other hand, cluster analysis is used to find groups of individuals with similar symptom profiles; it groups people instead of factors 22. No work has yet been done to determine the most suitable approach for finding symptom clusters; however, it is important for researchers to begin both to refine and to define the statistical method used in symptom cluster work.

The next step would be to select the assessment tools appropriate for data collection. Seven of the reported studies used 13 different symptom assessment tools; almost every study used a unique questionnaire. Because the analysis is based on the symptoms and scaling used in the assessment tools, it is important that similar assessment tools be used in all symptom cluster research. An 11-item questionnaire will produce results different from those originating with a 32-item symptom inventory. Although different populations may require slightly different questionnaires, a basic symptom inventory with a sufficient sample of symptoms applicable to all cancer patients should be applied in symptom cluster research to ensure consistent data analysis. Otherwise, the symptom clusters derived will vary depending on the symptoms listed in the assessment tools.

Symptom research will remain a complicated topic because of the many confounding factors in the symptom experience. Symptoms can be caused by the disease, the treatment, or even the comorbidities, and isolating the cause of each symptom is difficult. However, the exploration of symptoms within various demographic categories remains important. Once symptom cluster research has been refined, it will be useful to extract differences in symptom clusters by age, sex, cancer type, disease stage, and ethnicity.

Moreover, further work in defining the length of time that symptoms occur together before they are considered a symptom cluster may be necessary, as suggested by Barsevick 22. This distinction may be difficult, because symptom clusters may evolve with the deterioration that occurs as cancer progresses. Nevertheless, the question is valid and necessary in investigating symptom clusters.

Symptom research and the definition of symptom clusters in the oncology population will revolutionize treatment and diagnosis. The discovery of validated symptom clusters will aid diagnostic criteria, assessment, management, and prioritization of care. Many papers have confirmed the prognostic effect of multiple symptoms and the effect of those symptoms on quality of life and functional status 16,25–29. Thus the study of symptom clusters has important implications in treatment strategy and understanding of cancer.

## Figures and Tables

**TABLE I:** Review of symptom clusters in cancer patients^5^–^11^

Reference	Patients (*n*)	Cancer site	Statistical analysis method	Symptom cluster	Associated symptoms	Assessment tools
Walsh and Rybicki, 2006 5	922	No specific site	Cluster analysis (agglomerative hierarchical with average linkage)	Fatigue/anorexia–cachexia Neuro-psychological Upper gi Nausea/vomiting Aerodigestive Debility Pain	Fatigue, weakness, anorexia, lack of energy, dry mouth, early satiety, weight loss, taste change Sleep, depression, anxiety Dizziness, dyspepsia, belching, bloating Nausea/vomiting Dyspnea, cough, hoarseness, dysphagia Edema, confusion Pain constipation	38-item symptom checklist
Chen and Tseng, 2006 6	151 (23 outpatients) (128 inpatients)	No specific site	Factor analysis (principal-axis factoring with oblique rotation)	Sickness	Fatigue, sleep disturbance, lack of appetite, drowsiness	13-item MD Anderson Symptom Inventory, 14-item Hospital Anxiety Items and Depression scale
				Gastrointestinal	Nausea, vomiting	
				Emotional	Distress and sadness	
Wang *et al.*, 2004 8 (Chinese validation)	249	No specific site	Factor analysis (principal axis factoring with direct oblimin rotation)	General	Pain, fatigue, disturbed sleep, emotional distress, shortness of breath, drowsiness, dry mouth, sadness, difficulty remembering, numbness or tingling	MD Anderson Symptom Inventory
				Gastrointestinal	Nausea, vomiting	
Cleeland *et al.*, 2000 10 (English validation)	527	No specific site	Factor analysis (principal axis factoring with direct oblimin rotation)	General	Pain, fatigue, disturbed sleep, emotional distress, shortness of breath, drowsiness, dry mouth, sadness, difficulty remembering, numbness or tingling	MD Anderson Symptom Inventory
				Gastrointestinal	Nausea, vomiting	
Wang *et al.*, 2006 7 (Filipino validation)	206	No specific site	Factor analysis (principal axis factoring with direct oblimin rotation)	General	Pain, fatigue, disturbed sleep, emotional distress, shortness of breath, drowsiness, dry mouth, sadness, difficulty remembering, numbness or tingling	MD Anderson Symptom Inventory
				Gastrointestinal	Nausea, vomiting	
Okuyama *et al.*, 2003 9 (Japanese validation)	252	No specific site	Factor analysis (principal axis factoring with direct oblimin rotation)	General	Pain, fatigue, disturbed sleep, emotional distress, shortness of breath, drowsiness, dry mouth, sadness, difficulty remembering, numbness or tingling	MD Anderson Symptom Inventory
				Gastrointestinal	Nausea, vomiting	
Ivanova *et al.*, 2005 11 (Russian validation)	226	No specific site	Factor analysis (principal axis factoring with direct oblimin rotation)	General	Pain, fatigue, disturbed sleep, drowsiness, loss of appetite	MD Anderson Symptom Inventory
				Treatment-related	Nausea, vomiting, shortness of breath, numbness, difficulty remembering, dry mouth	
				Affective	Emotional distress, sadness	

**TABLE II:** Review of symptom clusters in lung cancer patients^12^,^13^

Reference	Patients (*n*)	Cancer site	Statistical analysis method	Symptom cluster	Associated symptoms	Assessment tools
Sarna and Brecht, 1997 12	60	Advanced lung (women)	Factor analysis (principal components with varimax rotation)	Emotional/physical suffering Gastrointestinal distress Respiratory distress Malaise	Pain frequency, pain severity, bowel, appearance, outlook Nausea frequency, nausea severity, appetite Insomnia, breathing, cough Fatigue, concentration	Symptom Distress Inventory
Gift *et al.*, 2004 13	220	Lung	Factor analysis	Cluster 1	Fatigue, weakness, nausea, vomiting, loss of appetite, weight loss, altered taste	37-item Physical Symptom Experience tool

**TABLE III:** Review of symptom clusters in breast cancer patients^14^–^16^

Reference	Patients (*n*)	Cancer site	Statistical analysis method	Symptom cluster	Associated symptoms	Assessment tools
Bender *et al.*, 2005 14	40 Early stage	Breast	Hierarchical cluster analysis	Fatigue Perceived cognitive impairment Mood problems Other	Fatigue, lacking energy, weakness Memory problems, concentration loss Anxiety, depression Difficulty sleeping aching muscles, backaches	Profile of Mood States, Symptom Checklist, daily symptom diary, Menopausal Quality of Life scale, Functional Assessment of Cancer Therapy Anemia/Fatigue scale
	88 Stage i/ii/iii			Fatigue Perceived cognitive impairment Mood problems	Fatigue, lacking energy, weakness Memory problems, concentration loss Anxiety, depression	
	26 Stage iv			Fatigue Mood problems	Fatigue, lacking energy, weakness Anxiety, depression	
	TOTAL: 154		Common clusters:	Fatigue Mood problems		
Glaus *et al.*, 2006 15	373 (301 early stage) (72 late stage)	Breast (hormone treatment)	Cluster analysis (agglomerative hierarchical method)	Menopausal	Hot flashes/sweats, weight tiredness/fatigue, gain, vaginal dryness and decreased sexual interest	Clinical Checklist for Patients with Endocrine Therapy, International Breast Cancer Study Group Linear Analogue Scales for patients with endocrine treatment
Ridner, 2005 16	64	Breast (lymphedema)	ANCOVA	Cluster 1	Alteration in limb sensation, loss of confidence in body, decreased physical activity, fatigue, psychological distress	52-Item symptom checklist, 11-item Iowa 11×3 short-form Center for Epidemiologic Studies of Depression, 37-item Profile of Mood States—Short Form

## References

[1] Chang VT, Hwang SS, Feuerman M, Kasimis BS (2000). Symptom and quality of life survey of medical oncology patients at a veteran affairs medical centre: a role for symptom assessment. Cancer.

[2] Portenoy RK, Thaler HT, Kornblith AB (1994). Symptom prevalence, characteristics and distress in a cancer population. Qual Life Res.

[3] Dodd MJ, Miaskowski C, Paul SM (2001). Symptom clusters and their effect on the functional status of patients with cancer. Oncol Nurs Forum.

[4] Kim HJ, McGuire DB, Tulman L, Barsevick AM (2005). Symptom clusters: concept analysis and clinical implications for cancer nursing. Cancer Nurs.

[5] Walsh D, Rybicki L (2006). Symptom clustering in advanced cancer. Support Care Cancer.

[6] Chen ML, Tseng HC (2006). Symptom clusters in cancer patients. Support Care Cancer.

[7] Wang XS, Laudico AV, Guo H (2006). Filipino version of the M.D. Anderson Symptom Inventory: validation and multisymptom measurement in cancer patients. J Pain Symptom Manage.

[8] Wang XS, Wang Y, Guo H, Mendoza TR, Hao XS, Cleeland CS (2004). Chinese version of the M.D. Anderson Symptom Inventory: validation and application of symptom measurement in cancer patients. Cancer.

[9] Okuyama T, Wang XS, Akechi T (2003). Japanese version of the M.D. Anderson Symptom Inventory: a validation study. J Pain Symptom Manage.

[10] Cleeland CS, Mendoza TR, Wang XS (2000). Assessing symptom distress in cancer patients: the M.D. Anderson Symptom Inventory. Cancer.

[11] Ivanova MO, Ionova TI, Kalyadina SA (2005). Cancer-related symptom assessment in Russia: validation and utility of the Russian M.D. Anderson Symptom Inventory. J Pain Symptom Manage.

[12] Sarna L, Brecht ML (1997). Dimensions of symptom distress in women with advanced lung cancer: a factor analysis. Heart Lung.

[13] Gift AG, Jablonski A, Stommel M, Given CW (2004). Symptom clusters in elderly patients with lung cancer. Oncol Nurs Forum.

[14] Bender CM, Ergyn FS, Rosenzweig MQ, Cohen SM, Sereika SM (2005). Symptom clusters in breast cancer across 3 phases of the disease. Cancer Nurs.

[15] Glaus A, Boehme Ch, Thurlimann B (2006). Fatigue and menopausal symptoms in women with breast cancer undergoing hormonal cancer treatment. Ann Oncol.

[16] Ridner SH (2005). Quality of life and a symptom cluster associated with breast cancer treatment–related lymphedema. Support Care Cancer.

[17] Ballatori E, Roila F (2003). Impact of nausea and vomiting on quality of life in cancer patients during chemotherapy. Health Qual Life Outcomes.

[18] Glaus A, Knipping C, Morant R (2004). Chemotherapy-induced nausea and vomiting in routine practice: a European perspective. Support Care Cancer.

[19] Beck SL, Dudley WN, Barsevick A (2005). Pain, sleep disturbance, and fatigue in patients with cancer: using a mediation model to test a symptom cluster. Oncol Nurs Forum.

[20] Fleishman SB (2004). Treatment of symptom clusters: pain, depression, and fatigue. J Natl Cancer Inst Monogr.

[21] Miaskowski C, Lee KA (1999). Pain, fatigue, and sleep disturbances in oncology outpatients receiving radiation therapy for bone metastasis: a pilot study. J Pain Symptom Manage.

[22] Barsevick AM, Whitmer K, Nail LM, Beck SL, Dudley WN (2006). Symptom cluster research: conceptual, design, measurement, and analysis issues. J Pain Symptom Manage.

[23] Miaskowski C, Dodd M, Lee K (2004). Symptom clusters: the new frontier in symptom management research. J Natl Cancer Inst Monogr.

[24] Thompson BM (2004). Exploratory and Confirmatory Factor Analysis: Understanding Concepts and Applications.

[25] Teunissen SC, Graeff A, de Haes HC, Voest EE (2006). Prognostic significance of symptoms of hospitalised advanced cancer patients. Eur J Cancer.

[26] Walsh D, Rybicki L, Nelson KA, Donnelly S (2002). Symptoms and prognosis in advanced cancer. Support Care Cancer.

[27] Dodd MJ, Miaskowski C, Paul SM (2001). Symptom clusters and their effect on the functional status of patients with cancer. Oncol Nurs Forum.

[28] Fox SW, Lyon DE (2006). Symptom clusters and quality of life in survivors of lung cancer. Oncol Nurs Forum.

[29] Miaskowski C, Cooper BA, Paul SM (2006). Subgroups of patients with cancer with different symptom experiences and quality-of-life outcomes: a cluster analysis. Oncol Nurs Forum.

